# The economic impact of the Covid-19 pandemic on Sri Lankan migrants in Qatar

**DOI:** 10.1186/s40878-021-00246-0

**Published:** 2021-08-30

**Authors:** Anoji Ekanayake, Kopalapillai Amirthalingam

**Affiliations:** 1 The International Centre for Ethnic Studies, Colombo, Sri Lanka; 2 Department of Economics, University of Colombo, Colombo, Sri Lanka

**Keywords:** Covid-19, Pandemic, Migration, GCC region, Qatar, Sri Lanka

## Abstract

The spread of Covid-19 in Qatar and the pandemic-led economic slump in the country have substantial financial implications for Sri Lankan migrant workers in Qatar and the Sri Lankan economy as a whole, as Qatar has been one of the primary destinations among Sri Lankan migrants in recent years. Based on 12 in-depth interviews and an online survey of 101 Sri Lankan workers in Qatar, this paper assesses the pandemic’s financial implications on three groups of Sri Lankan migrants; the highly-skilled, skilled and semi-skilled. Using a mixed-method analysis, the paper identifies that pay-cuts have been the most common financial issue across all skill levels, with nearly half of respondents reporting deductions from their salaries. The research also identifies that while all three groups of migrants have used various coping mechanisms to mitigate the pandemic’s financial impact, highly-skilled migrants have been more successful in weathering the storm than others due to their accumulated savings. Though compared to the early months of the pandemic, the financial stability of most Sri Lankans in Qatar had improved by September 2020 with the easing of restrictions imposed to contain the spread of Covid-19, it might not necessarily transfer into stability in remittances to Sri Lanka, as an increasing number of Sri Lankan migrant workers in Qatar are considering a permanent return home.

## Introduction

As the Gulf War of 1990, the global financial crisis in the late 2000s, floods in Thailand in 2011 and numerous other natural and human-made crises indicate, at times of crises in host countries, migrant workers tend to be disproportionately affected compared to native-born workers and face extreme hardships. Among other factors, their limited understanding of local languages, lack of support and resources received, discriminatory policies implemented to protect native workers, and in the case of irregular migrants, issues pertaining to their legal status in the host countries make them particularly vulnerable at times of strife and disasters in their host nations (Alessandra Bravi et al., 2017; Hendow, 2020; Weeraratna, 2020). Therefore, the Covid-19 pandemic, which has drastically changed the lives of billions of people since the beginning of 2020, has been particularly challenging for migrant workers, most of whom were in vulnerable situations even before the onset of the pandemic.

Among various groups of migrant workers, those in the Gulf Corporation Council (GCC)^1^ region have also been affected by the pandemic since its onset in early 2020. Even though the GCC region has not seen as many cases of Covid-19 as regions such as Europe, Northern America and Latin America, migrant workers in the GCC, who account for an average of 70% of the region’s labour force (Equidem, 2020), have faced severe financial and psycho-social issues in addition to the obvious health risk of the pandemic.

At the onset of the pandemic, the cramped labour camps where most low-wage migrant workers in the Gulf reside became breeding grounds for Covid-19 and had to be placed in isolation with entry and exit barred. As the months passed by, due to lockdowns and other measures imposed to contain the spread of Covid-19 and the pandemic-led drastic decline in oil prices in the world market have contracted the GCC economies resulting in lay-offs, pay-cuts, delays in payments and other financial issues for migrants belonging to all skill levels (Ekanayake, 2020). While the GCC countries implemented stimulus packages to help their populations affected by the pandemic, most of these have been geared towards benefiting only citizens and local businesses, and migrant workers have been given limited financial support (Equidem, 2020). As a result, these migrants and their families that depend on the remittances they send home have been severely affected by the pandemic.

While a few reports have been already published on how the pandemic has affected migrant workers in the GCC (such as those by Equidem (2020), Weeraratna (2020), World Bank Group (2020) and KPMG Qatar (2020)), these have either looked at instances where migrant workers’ human rights have been violated or the macroeconomic impact. To the best of our knowledge, none of the existing studies on the impact of the pandemic on migrant workers in the GCC has provided a thorough examination of how the pandemic has affected the migrant workers on a personal level and their struggles to take care of their families back home. Hence, this paper that assesses the economic impact of the pandemic on Sri Lankan migrant workers in Qatar contributes to the literature in many ways. First of all, it compares the financial burden of the pandemic on migrant workers belonging to three skill levels; the highly-skilled, skilled and the semi-skilled. Secondly, it examines the changes in the financial stability of migrant workers as the pandemic progressed by comparing the situation in the first trimester (March–May 2020) of the pandemic with that of the second trimester (June–August 2020). Thirdly, the paper assesses the coping mechanisms utilised by migrant workers to address their financial difficulties posed by the pandemic. Finally, it examines the extent to which the pandemic has influenced migrant workers’ return decisions. These observations would strengthen the literature on migration and remittances during crises, particularly the literature on remittances and return migration from the GCC to South Asia during times of strife, which is currently sparse.

The rest of the paper is organised as follows. The next section outlines how the Covid-19 pandemic unfolded in Qatar and the pandemic’s impact on the country’s migrant population. This is followed by the section that deals with the empirical literature on migration and remittances during disasters and other crises. The fourth section is on the data and methodology, followed by the section on the financial issues encountered by Sri Lankan workers in Qatar, where we compare the situation at the onset of the Covid-19 pandemic in March 2020 with that of the situation in September 2020. Then we examine the coping mechanism used by Sri Lankan migrant workers affected the pandemic and how the pandemic has affected the long-term plans of Sri Lankans in Qatar. Finally, we provide some concluding remarks.

## Unfolding the Covid-19 pandemic in Qatar

The first case of Covid-19 in Qatar was reported on 29 February 2020 (KPMG Qatar, 2020), and by mid-March, it developed into a massive outbreak with the spread of the disease in cramped labour camps in the Doha Industrial Area, where hundreds of thousands of low-wage migrant workers reside at (Pattison & Sedhai, 2020). Even though, in the beginning, the spread of Covid-19 in Qatar was mainly concentrated in the Industrial Area, it soon became rampant in all parts of the country. By July 2020, Qatar had reported over 100,000 cases of Covid-19 and was the country with the second-highest number of Covid19 cases in the Gulf region and the highest per capita rate of Covid19 in the world (Gambrell, 2020).

With the outbreak of Covid-19 in March, the Government of Qatar implemented the Covid-19 Qatar National Response Action Plan with the aims of enabling the society to function as normally as possible during and after the pandemic, minimising the economic impact of the pandemic and mitigating the impact of the pandemic on people of Qatar (Ministry of Public Health Qatar, 2020). Due to the various containment measures undertaken combined with the country’s young population, even though Qatar had a high number of reported cases, its fatality rate remained less than 0.1% of total cases in 2020, one of the lowest in the world (Varley, 2020).

While the country has done relatively well from a health perspective, the Qatar economy, akin to other Gulf economies, has been severely affected by the pandemic. International Monetary Fund (IMF) predicted that Qatar’s economy would contract by 4.3 % in 2020, the highest slip in the GCC region (International Monetary Fund, 2020). One of Qatar’s main pandemic-induced economic issues is the unprecedented drastic reduction in global demand for petroleum and gas. With factories operating far below the optimal capacity and fewer vehicles on the road due to lockdowns imposed all around the world, the demand for oil and gas, which were already in decline for the last six years due to a slowdown in China and other developing economies has experienced a severe drop. As the hydrocarbon sector accounts for 48% of Qatar’s GDP (Qatar National Bank, 2018), the decline in oil and gas prices has a massive impact on Qatar’s economy (KPMG Qatar, 2020).

Apart from the oil and gas sector, Qatar’s non-hydrocarbon sector, which has shown significant growth in recent years, was severely affected by the pandemic, especially in the early months when most economic activities in the country were halted. Among non-hydrocarbon sectors, the construction sector, which accounts for around 9 % of the country’s GDP and is considered the driving force of the non-hydrocarbon growth (Qatar National Bank, 2018), has been particularly affected. While the government sector projects mostly related to the 2022 FIFA World Cup have not been affected by the pandemic, private sector constructions projects have been greatly affected (KPMG Qatar, 2020). Lockdown of the industrial area from mid-March to early May 2020, where many construction workers and materials for construction sites are located, disrupted many construction projects (Collier, 2020). Moreover, as the construction industry in Qatar is heavily dependent on overseas suppliers for materials, the spread of the pandemic in other countries, particularly in Europe and the United States from where mechanical, electrical and plumbing products are imported from could affect the timelines of construction projects increasing costs (Collier, 2020). Apart from construction, other non-hydrocarbon sectors severely affected by the pandemic are transportation, warehousing, retail trade, accommodation, and leisure services (Omar, 2020).

In order to mitigate the economic impact of the pandemic on Qatar economy, in mid-March 2020, Qatar’s Supreme Committee for Crisis Management (SCCM) declared Qatar Riyal 75 billion (USD 20.5 billion) stimulus package to the private sector while the Qatar Central Bank reduced interest rates in order to provide additional liquidity to banks to improve lending conditions. Furthermore, the Government of Qatar increased its investments in the Qatar Stock Exchange by Qatar Riyal 10 billion to boost the market (Qatar Tackles Coronavirus with Stimulus Package, 2020). Even though these measures have been able to mitigate the adverse economic impact to a certain extent, the country’s gross domestic product fell by 6.1% in the second quarter of 2020 (Omar, 2020). However, with the gradual lifting of the lockdown since 15 June 2020, the country’s economic activities began to bounce back in the third quarter of 2020.

## Impact of the pandemic on Qatar’s migrant population

The economic downturn in Qatar in 2020 has had a severe negative impact on its migrant population which accounts for around 88% of the country’s workforce (Equidem, 2020). At the early stage of the pandemic, the migrants most affected were unskilled and semi-skilled workers, especially those residing in the cramped accommodations in the Doha Industrial Area, which was placed under lockdown for more than a month. However, as the pandemic began to spread and the economic activities in the country were halted, migrants belonging to all skill levels began to feel the economic impact of the pandemic. For example, by June, Qatar Petroleum, the world’s largest producer of liquefied gas, slashed the jobs of around 800 migrant workers while Qatar Airways, one of the main employers of the country, terminated around 9000 foreign workers (HRK News Bureau, 2020; Tahir & Bhatti, 2020).

While the health impact of the pandemic was felt by both Qatari citizens and foreign guest workers, when it comes to the economic impact, in most sectors, migrants had to bear the brunt of the pandemic. For instance, in early June, Qatar’s Ministry of Finance instructed all entities that are 100% state-funded to cut spending on its foreign employees by 30% either through pay cuts or layoffs (Abadi, 2020;Foxman, 2020 ; HRK News Bureau, 2020) while for Qatari citizens only their allowances were slashed (Abadi, 2020). However, layoffs and pay cuts of foreign workers could adversely affect Qatar’s economy due to decreased consumer spending and labour shortages. It has been predicted that there will be an exodus of 10% of Qatar’s population due to the pandemic, which could have a pervasive long-lasting impact on the country’s economy (Foxman, 2020; HRK News Bureau, 2020).

## Migration and remittances during crises

Migrant workers are affected by disasters and calamities both in their home and host countries. While they are, in general, tuned to most crises in their countries of origin, they become particularly vulnerable in the face of crises in their host countries due to a lack of supportive structures and discriminatory policies that favour native-born workers over migrants (Alessandra Bravi et al., 2017; Hendow, 2020; Weeraratna, 2020). Among other crises such as natural disasters and conflicts, severe economic downturns in host countries particularly affect migrant workers as the brunt of such economic crises are felt not only by them but their dependents in their home countries as well.

Financial crises in host countries can lead to increasingly unpredictable and sometimes hazardous working conditions, reduced work hours, pay cuts, breaches in contracts, and employer bankruptcies resulting in downsizing, leaving some migrant workers destitute and indebted to individuals and loaning agencies for money borrowed to cover costs of migration and work permits (Spitzer & Piper, 2014). During such times, migrant workers struggling to make their ends meet find it challenging to send remittances to their dependents in their home countries. For instance, during the Global Financial Crisis (GFC) in the late 2000s, many migrant workers in the GCC lost their jobs and had to cut down their consumption and resort to coping mechanisms such as sharing accommodations to save money to remit to their families back home (Sirkeci et al., 2012). In addition, data suggest that migrants who are severely affected by crises in host countries may resort to using their savings back home (i.e. reverse remittances) to sustain their lives in the host country. For instance, it is assumed that the GFC forced migrants from the Dominican Republic in the United States to utilise their savings in the Dominican Republic (Ratha, 2009).

While there is limited research on remittance patterns of migrants during crises in their adopted nations, studies on the relationship between crises and remittances suggest that remittances increase in the aftermath of disasters and crises in the countries of origin (Mohapatra et al., 2011) due to altruistic, selfish and inter-temporal motivations of migrants (Clarke & Wallsten, 2003).^2^ Empirical data on remittances at times of crises in home countries shows that remittances increase and remain stable after severe shocks such as natural disasters, financial crises and armed conflicts in migrants’ home countries (Clarke & Wallsten, 2003; Mohapatra et al., 2011; Yang, 2006). For instance, after the floods in Bangladesh in 1998, the tsunami in Sri Lanka in 2004 and Hurricane Ivan in Grenada in 2004, remittances received by these countries significantly increased (Savage & Harvey, 2007).

Remittances play an important role in the aftermath of disasters as they represent a relatively stable form of income for a country affected by a crisis. The stability of remittances is based on the fact that remitters, in general, are unlikely to be affected by the same crisis as their families back home as they reside overseas. For instance, while the 2004 tsunami-affected Sri Lanka, the country continued to receive remittances even at a higher rate than before the disaster as most Sri Lankan migrant workers reside in the GCC, a region unaffected by the tsunami (Savage & Harvey, 2007). Even though crises that affect both home and host countries are rare, they do occur, and during such times, remitting home can be difficult for migrant workers who themselves might be struggling to survive in their host countries (Savage & Harvey, 2007). However, research on the remittance behaviours of migrant workers during crises that affect both home and host countries is sparse.

Financial difficulties and the inability to remit are not the only possible outcomes of crises in host countries. At times, unemployment and other vulnerabilities that migrants face during crises in host countries can motivate or force them to return to their home countries. For instance, the Asian financial crisis in 1997 expedited the return of Bangladeshi migrant workers in Southeast and East Asian nations as they found it difficult to renew their work permits or were deported back to Bangladesh (Ahmed, 1998). During the GFC in the late 2000s, countries such as Malaysia, Singapore, Thailand, and South Korea attempted to limit certain forms of labour migration, return migrant workers, and increase deportations of irregular migrants to reduce pressure on the native population (Spitzer & Piper, 2014). However, despite such measures, crises in host nations may not always lead to a massive outflow of migrant workers. For instance, during the GFC, some migrant workers were unwilling to return home as they feared re-entry to their host nations would be difficult once the crisis was over (Sirkeci et al., 2012). However, there is limited literature on the factors that affect the return decisions of migrants during crises in host countries to determine the extent to which this applies to various host nations.

As the literature on how crises affect migrant workers is sparse, further empirical data is needed to examine the financial decision making of migrant workers during crises. Therefore, this paper, which examines financial difficulties faced by Sri Lankan migrant workers in Qatar due to the Covid-19 pandemic, a crisis that has affected both host and home countries, addresses a significant gap in the existing literature on migrants during crises. The paper particularly seeks to address the gap on how crises in host countries may affect migrants’ coping mechanisms, remittances they send home and their return migration decisions.

## Methodology

### Selecting the study population

Qatar is home (although a temporary one) to migrant workers belonging to more than 90 nationalities (Priya Dsouza Communications, 2019). Among them, the researchers selected Sri Lankans as the study population due to two main reasons. Firstly, Sri Lankans are among the top ten nationality groups in Qatar,^3^ and in recent years Qatar has been the primary destination among Sri Lankan migrant workers to the GCC, with nearly a quarter of all Sri Lankan migrant workers migrating to Qatar in 2018 (SLBFE, 2019). According to the Sri Lankan Embassy in Qatar, as of 2018, approximately 140,000 Sri Lankans resided in Qatar (Asees, 2018). The second reason for selecting Qatar is that one of the researchers is a Sri Lankan migrant currently based in Qatar. Her first-hand experience of how the pandemic unfolded in Qatar and how it affected the country’s migrant workers, particularly Sri Lankan migrant workers, were used in designing the survey. Moreover, both researchers have done prior research on Sri Lankan migrant workers in Qatar and have a network of migrant workers that is easily accessible. These prior connections were critical when conducting fieldwork during a pandemic when face-to-face interactions were not plausible.

## Research instruments

This study is solely based on primary data gathered through two instruments: semi-structured in-depth interviews with 12 participants conducted over the phone and a web-based survey of 101 respondents.

### In-depth interviews

The in-depth interviews were conducted in two segments. The first batch of interviews, which included three interviews, was conducted in May 2020 before designing the survey. The second group of interviews were conducted in September 2020, two months after the survey data was gathered. While the first batch of interviews was used to design the survey, the data from both batches of interviews were used to assess the survey results.

All interview participants were recruited using purposive sampling to ensure that the interviews covered various experiences of migrants belonging to different skill levels and varying levels of impact of the pandemic.

The interviewees were asked a range of open-ended questions about their migration history, aims and motivations of migration, comparison of life in Qatar prior to and post-pandemic. In addition, the interviews particularly looked at how the impact of the pandemic changed as the year 2020 progressed, the coping mechanisms adopted by the migrants in response to the financial pressures they were facing, whether the pandemic has led to changes in remitting and their plans regarding their return to Sri Lanka.

### Online survey

Internet users among migrants tend to be very high as the internet is a cheaper mode of communication with families in home countries. Therefore, in recent years, web-based surveys are increasingly becoming popular and commonplace among migration researchers. Online surveys tend to provide easy access to migrant groups, and their anonymous nature leads to better inclusion of irregular migrants (Mieriņa, 2019). Due to these benefits of online surveys, as well as to comply with social distancing rules imposed to combat the spread of Covid-19 in Qatar, a web-based survey was utilised for the present study.

The online questionnaire was developed based on the data collected from the first segment of interviews and was conducted between 3 and 15 June 2020. The questionnaire was kept short to receive a higher rate of responses, and was pre-tested on five participants. The survey included questions regarding the financial difficulties the respondents were facing at the time of the survey and the challenges they expect to encounter in the near future. In addition, the survey included questions regarding how the pandemic has affected the remittances sent to Sri Lanka and whether the pandemic has affected the migrant workers’ plans regarding the length of their stay in Qatar.

Since a list of all members of the population was not available, the sample for the questionnaire was selected using the snowball sampling method. The survey was initially sent to Sri Lankan migrant workers in Qatar whom the researchers had contacted for their prior studies in Qatar. In addition, the survey was shared in several formal and informal WhatsApp groups maintained by Sri Lankan professional associations such as the ones maintained by the Institute of Engineers Sri Lanka and Sri Lanka Quantity Surveyors in Qatar. The survey was also shared on Facebook pages and groups for Sri Lankan migrant workers in Qatar. Apart from these, the researchers asked their friends and acquaintances in Qatar to share the survey among their Sri Lankan friends.

While the data collected through online surveys can be skewed due to selection biases, the researchers tried the maximum to avoid this with the current data set by making the survey available in all three languages (Sinhala, Tamil and English) spoken by Sri Lankans and sharing the survey with Sri Lankan migrant workers with varying experiences of the financial impact of the pandemic ranging from high impact to no impact at all. In addition, the survey was sent out to participants working in various organisations and residing in locations spread across Doha. However, due to limitations of the snowball sampling method, a significant proportion of high-skilled workers who filled out the survey were engineers, quantity surveyors and managers, while the majority of semi-skilled workers were vehicle mechanics and drivers. Moreover, the researchers could not recruit any unskilled workers to participate in the survey. This caveat has skewed the results and analysis towards the experiences of certain occupational and skill categories and is the main limitation of the study.

All the data gathered were used in a mixed-method analysis to gain more precise in-depth insights into the financial impact of the Covid-19 pandemic on various groups of Sri Lankan migrant workers in Qatar. The data from the in-depth interviews were used in thematic qualitative analysis, whereas the information collected through the online survey was analysed using quantitative methods with the aid of Microsoft Excel. In instances where interviewees’ experiences are used as case studies, pseudonyms are used to refer to them to protect their identities.

## Descriptive data

Among the survey participants, 82% were males, and 18% were females. The majority (67%) of the respondents were between the ages of 31–40, while 15% were 21–30 years old. Another 15% were in the age group of 41–50, while only 4% were above 50 years old. The majority of the survey respondents were married with families residing with them in Qatar (57.4%) or Sri Lanka (30.7%). The rest were either single, divorced or separated. Among the interviewees, nine were male, while the other three were females. Ten were in the 31–40 age group while the other two were in the 21–20 and 41–50 age groups. Eleven of the interviewees were married, while the other one was single.

The twelve in-depth interview participants included six highly skilled workers, three skilled workers and three semi-skilled workers. Among survey participants, 78 % were highly-skilled, 14% were semi-skilled, and 9 % were skilled. Neither the in-depth interviews nor the survey includes any unskilled migrant workers, and hence the present analysis focuses only on the experiences of migrants in the highly-skilled to semi-skilled spectrum.

The International Standard Classification of Occupations (ISCO) of the International Labour Organisation (ILO) (2012) was used to categorise both interviewees and survey participants based on their skill levels. ISCO classification includes four skill levels. Level-one jobs involve performing simple, routine, physical or manual tasks. Level-two occupations involve tasks such as driving vehicles, operating or maintenance of machinery and clerical work. Level three occupations involve performing complex technical and practical tasks that require knowledge in a specialised field and includes jobs such as shop managers, laboratory technicians, and legal secretaries. Level four occupations involve jobs requiring complex problem solving, decision-making, and creativity based on extensive knowledge in a specialised field. Jobs such as sales and marketing managers, engineering, medical practitioners and system analysts fall into this category (International Labour Organisation, 2012). For ease of understanding, this study refers to ISCO skill level one as unskilled, skill level two as semi-skilled, skill level three as skilled and skill level four as highly skilled.

## Results and discussions

### Financial impact of the COVID19 pandemic on the Sri Lankan migrants in Qatar

Through in-depth interviews and survey data we found that layoffs and pay cuts and Qatar’s overall economic decline due to the pandemic affected a significant percentage of Sri Lankan migrants in the country. At the time of the survey in June 2020, 67% of the respondents (*N* = 97) were faced with one or more financial issues due to the Covid-19 pandemic (Table 1). The most common problem reported was pay cuts, with nearly 50% of the survey participants stating that their salaries were slashed in the March–May 2020 period. The majority (61%) of those who were experiencing pay cuts did not think that they would receive the withheld salaries, while 35% were not sure whether they would be compensated when their companies bounce back post-pandemic. In-depth interviews revealed that even though pay cuts were considered a significant financial burden by Sri Lankan migrant workers as most of them had migrated to Qatar mainly because of the attractive remuneration packages the country offer, most of them considered reduced pay acceptable due to the volatile economic conditions in Qatar posed by the pandemic. Moreover, they were willing to accept a reasonable pay-cut rather than their companies running into bankruptcy which will push them to unemployment, exacerbating their financial problems.

**Table 1:** Type of financial difficulties faced by Sri Lankan migrants in Qatar

Type of financial difficulty faced	All	Highly-skilled	Skilled	Semi-Skilled
Pay cuts	49.5%	45.9%	66.7%	57.1%
Delays in salary payments	19.6%	18.9%	33.3%	14.3%
Loss of job	9.3%	8.1%	11.1%	14.3%
Loss of gratuity	2.1%	2.7%	0%	0%
Loss of allowances and bonuses	9.3%	8.1%	11.1%	14.3%
Loss of savings	24.7%	21.6%	33.3%	35.7%
Difficulty to remit to Sri Lanka	16.5%	12.2%	11.1%	42.9%
Difficulty to repay debts	20.6%	13.5%	11.1%	64.3%
Difficulty to pay rent	12.4%	14.9%	0%	7.1%
Difficulty to cover food and other basic expenses	9.3%	6.8%	0%	28.6%
No financial difficulties	33.0%	39.2%	11.1%	14.3%

Source: Survey data (June 2020)

Through survey data and in-depth interviews we found that there are considerable differences in the level and type of financial difficulties experienced across the skill levels. As can be expected, the skilled and semi-skilled workers who, in general, are in more vulnerable situations than high-skilled workers were faced with more financial difficulties than the highly-skilled due to the pandemic. According to survey data, whereas 39% of highly-skilled workers stated that they are yet to face any financial difficulty due to the pandemic, only 11% of skilled workers and 14% of semi-skilled workers reported they were yet to encounter any financial pressures due to the pandemic (Table 1).

As can be expected, survey data indicate that semi-skilled workers experienced the most difficulties sending remittances to Sri Lanka, paying off their debts and managing their food and other expenses. Compared to highly-skilled workers, a higher percentage of skilled and semi-skilled Sri Lankans in Qatar have experienced pay cuts, layoffs, losses in bonus payments, and losses in savings due to the pandemic. On the other hand, a higher percentage of highly-skilled Sri Lankans in Qatar have experienced losses in gratuity payments and have faced difficulties making rental payments than skilled and semi-skilled workers.

The higher percentage of semi-skilled workers experiencing difficulties in sending remittances during the early months of the pandemic could be partly because all in-person money exchange and transfer offices in Qatar were closed from 26 March to 11 May 2020 as part of measures undertaken by the Government of Qatar to contain the spread of Covid-19. This could have created difficulties for semi-skilled workers, who unlike skilled and highly-skilled workers, lack access and technological know-how to use online methods for remitting.

According to interviewees, compared to Sri Lankan highly-skilled and skilled workers in Qatar, semi-skilled workers tend to struggle more with debt repayments. In general, a higher percentage of semi-skilled workers than highly-skilled and skilled workers tend to obtain loans in Sri Lanka to finance their migration projects. Hence, these semi-skilled workers spend months and sometimes years paying off their debts which tend to be a substantial proportion of their salaries (Equidem, 2020). Therefore, even minor pay cuts and bonus losses could push them into a debt spiral, making it difficult for them to bear the living expenses in Qatar. Even though in March 2020, the Government of Sri Lanka provided a six-month moratorium on loans issued by banks in Sri Lanka to provide relief to those who have lost income due to the pandemic (Relief Measures to Assist Covid-19 Affected Businesses and Individuals, Pub. L. No. No 04 of 2004, 2020), such measures seem to have not been beneficial for semi-skilled workers. This is because most semi-skilled workers tend to rely on informal loan schemes such as borrowing from local money lenders.

On the other hand, the rental issues experienced by the highly-skilled could be due to the nature of rental agreements of accommodations where the highly-skilled workers tend to reside. Usually, highly-skilled workers in Qatar live in apartment complexes with binding contracts and hence might find it difficult to move out with short notice despite facing pay cuts and delays in payments. As semi-skilled and skilled workers are usually either provided with accommodation by their companies or stay in places that are more flexible about moving out, they might not have experienced issues with rental payments as much as highly-skilled workers.

### Remittances to Sri Lanka

At the time of the survey in June 2020, approximately half (53%) the survey respondents (*N* = 101) stated that they had to decrease the amount of money they remit to Sri Lanka between March and May 2020, while 31% said that they did not make any changes to the amount of money sent to Sri Lanka. Only 16% of the respondents stated that they increased remittances to Sri Lanka during the same period. All those who increased their remittances to Sri Lanka are high-skilled workers, while all skilled and semi-skilled workers either decreased or made no changes to the amount sent to Sri Lanka.

The main reason for decreasing the amount of money sent to Sri Lanka (*N* = 54) was pay cuts (55.6%). This was followed by overall economic uncertainties created by the pandemic (38.9%), potential pay cuts (33.3%) and potential layoffs (24.1%) in the coming months. Temporary closure of money transfers offices (14.8%) and layoffs (9.3%) were among other reasons for decreasing the remittances to Sri Lanka.

On the other hand, the main reason of those who increased remittances to Sri Lanka (*N* = 16) was to support their friends and family members in Sri Lanka who were facing economic difficulties due to the pandemic (87.5%), followed by the desire to increase savings in Sri Lanka due to potential layoffs resulting in a permanent return to Sri Lanka in the coming months (25%).

Increasing remittances due to the desire to increase savings in Sri Lanka falls into the category of self-interest remittances, which is linked to migrants’ intention of returning to the home countries eventually (Arun & Ulku, 2011). Remitting to support family and friends could fall into the category of altruistic remittances where transfers are made to their home countries without expecting anything in return (at least in the immediate future). This is consistent with the New Economics of Labour Migration (NELM) theory which states that altruistic migrants would remit more money as their family encounters economic difficulties at home (Arun & Ulku, 2011; Lim & Basnet, 2017). However, those who remitted to support their families communities back home could have done it also due to expectations from their friends and family to help them out in times of need as Sri Lankans, in general, believe that Sri Lankans abroad have better financial resources than them and are hence obliged to support those left behind. This falls into the category of socially controlled remittances in the model introduced by Mahmud (2020) and is similar to the experiences of some of the Bangladeshi migrants in Tokyo who are coerced to remit due to emotional manipulations, social shaming and other informal pressures imposed by migrants’ families and communities.

### Changes in the pandemic-induced financial pressures

At the onset of the pandemic, most Sri Lankan workers in Qatar feared for their job security and anticipated their financial situation could be worsened in the coming months due to a pandemic-led economic slump in Qatar. Part of this fear was based on their experience of seeing their friends and colleagues losing their jobs due to the economic sanctions imposed on Qatar by the neighbouring Arab nations in 2017.^4^ Moreover, even before the onset of the pandemic, some of them had begun to experience a slowdown in their workload as a result of the decline in Qatar’s economic growth over the years.

The survey data also indicates that most respondents anticipated that their economic conditions would worsen as 2020 progressed. As opposed to the 67% of respondents who were already facing financial difficulties at that time of the survey in June 2020, 93% of respondents believed that they would encounter some form of financial difficulty in the near future (Table 2).

**Table 2:** Comparison of present and anticipated financial issues faced by Sri Lankan migrants in Qatar

Type of financial difficulty faced	The proportion of respondents currently faced with the financial issue	The proportion of respondents’ who expect they will face the financial issue in the near future
Pay cuts	49.5%	54.0%
Delays in salary payments	19.6%	31.0%
Loss of job	9.3%	42.0%
Loss of gratuity	2.1%	22.0%
Loss of allowances and bonuses	9.3%	36.0%
Loss of savings	24.7%	37.0%
Difficulty to remit to Sri Lanka	16.5%	19.0%
Difficulty to repay debts	20.6%	22.0%
Difficulty to pay rent	12.4%	19.0%
Difficulty to cover food and other basic expenses	9.3%	13.0%
No financial difficulties	33.0%	7.0%

Source: Survey data (June 2020)

The most common financial issue anticipated was pay cuts, with 54% of respondents expecting reductions in their salaries in the coming months. The most substantial difference between the present and anticipated financial difficulties was for the loss of gratuity payments, with 22% of respondents believing that they could lose their gratuity in the coming months as opposed to the 2% who had already lost their gratuity payments by the time of the survey in June 2020. This was followed by job losses, with 42% of respondents believing that they could lose their jobs in the coming months as opposed to the 9 % who had already lost their jobs by June.

However, in-depth interviews indicated that contrary to the expectation that the situation of Sri Lankan workers in Qatar would be worsened as the year progresses, by September 2020, the financial stability of most Sri Lankans had improved in comparison to the first few months of the pandemic. This can be explained through the case study of Vimal, a 39-year old senior manager of the events division of a floriculture company in Qatar. Vimal’s division which was already undergoing financial issues due to the economic sanctions imposed on Qatar by neighbouring Arab nations in 2017, first felt the impact of the pandemic in January 2020 when Qatar halted shipments from China as a Covid-19 containment measure.

“We were one of the first industries to feel the impact of the pandemic. When cargo from China was halted, we couldn’t get the necessary supplies. Then, because of Covid-19, people stopped buying flowers. So, our stores did not make a sufficient amount of sales. Our stores were completely shut between April and May. Since we are in the service sector, we were the ones who first faced the impact of Covid-19. The first full stop of Covid-19 was for us.”

Vimal states that even though he continued to pay the basic salaries of his employees in the first few months of 2020, by May 2020, he had to lay off or transfer his employees to other divisions of the company due to the weak financial status of his division. However, by September 2020, with the gradual lifting of Covid-19 prevention restrictions in Qatar, Vimal’s division began to receive new orders, and he has managed to rehire some of the staff on a part-time basis. Vimal says that he has now begun to feel “cautiously optimistic” about his business prospects.

In-depth interviews with other participants also revealed that the financial situation of most Sri Lankan migrants in Qatar has improved since June 2020. For some, the improvement in the financial status is because their salaries are no longer subjected to pay cuts. Even those who continue to face salary and bonus cuts consider their financial stability to have improved as their fears of being laid-off have somewhat subsided. For instance, Tharuki, a 32-year-old quantity surveyor, believes that with the gradual relaxation of Covid-19 containment related restrictions in Qatar and the departure of some laid-off migrants and those who wanted to be with their families during the pandemic, compared to the March–June period, there are more opportunities available for those who seek jobs in Qatar. She says that her fears about her job security have reduced as she now has to cover the workload of two former colleagues who were laid off due to the pandemic. She believes that her company cannot afford to fire her.

Those who continued to face significant economic hardships in September 2020 were mostly unskilled and semi-skilled workers who had lost or resigned from their jobs and were waiting to return to Sri Lanka. The visas of some of these migrants were expired, and hence they could not be hired by a new company. They could not return to Sri Lanka as they could not afford the hotel quarantine packages in Sri Lanka, which cost a minimum of LKR 100,000 (USD 500) and were beyond the reach of a laid-off semi-skilled migrant who used to work for a salary of QAR 1500–2000 (USD 400–550).^5^ Even though some of these stranded migrants were provided free accommodation and sometimes free meals from their former employers, they struggled as they found it difficult to manage other expenses and support their families in Sri Lanka.

### Coping mechanisms adapted by migrant workers

We found through in-depth interviews that akin to coping mechanisms adopted by South Asian migrant workers in the GCC due to the GFC in the late 2000s (Sirkeci et al., 2012), those faced with financial difficulties due to the pandemic resorted to borrowing, moving to accommodations with lower rentals and cutting down food and other expenses as coping mechanisms. For instance, Farooq, a 35-year-old automobile technician who has been stranded in Qatar without a job since April 2020, has cut down his food expenses and relies on his friends to support his family in Sri Lanka. Though his former employer allowed him to stay at his company-provided accommodations, without a job, he could not bear the expenses of his family in Sri Lanka nor afford his food expenses in Qatar. Farooq resorted to eating *kuboos* bread, an Arabic pita bread which costs 10 Qatar Dirhams (less than 1 USD cents). However, he still managed to send money to cover his family’s expenses in Sri Lanka, albeit the amount was 50% of what he used to remit. He says, “I don’t let my family feel the burden of Covid-19 at all. I balance everything so that they don’t feel the burden. I borrow from friends in Qatar and send money to Sri Lanka.”

Likewise, Asiri, a 38-year old skilled-worker involved in airlines operations, cut down his travel expenses to cope with the income loss due to pay cuts. Though Asiri’s financial situation was far better than most migrant workers in Qatar despite his 15% pay slash, he reduced his expenses to maintain his pre-pandemic remittances level to Sri Lanka. Asiri stated, “I have always remitted to Sri Lanka a fixed amount. That didn’t change because of Covid-19. It was because I cut down my expenditure in Qatar. For example, to reduce transportation expenses, I started going to places on foot rather than taking a taxi. I walk to the supermarket, which is around 2.5kms away from my home. Then, I come back with the groceries on foot instead of taking a taxi. I don’t want to reduce the amount I remit to Sri Lanka due to any external factor as I have to settle my housing loan in Sri Lanka.”

While cost-cutting to a certain extent can be expected in the face of any financial issue, we believe that some of these coping mechanisms which curtailed current expenditure in extreme ways are likely to be the result of severe financial conditions that migrant workers anticipated at the onset of the pandemic, as illustrated in Table 2. We found through in-depth interviews that though the situation of migrant workers improved by September 2020, they continued some of those cost-cutting practices either because their situation did not improve to a satisfactory level or because of their initial anticipated financial problems. It is worth exploring whether these migrant workers continued implementing some of these cost-cutting mechanisms even when their financial situation significantly improved.

Even though some Sri Lankan migrant workers in Qatar have been successful in coping with their financial struggles induced by the pandemic by cutting down their expenses, others have not been as lucky. Particularly, semi-skilled migrants who have lost their jobs and have been unable to return to Sri Lanka depend on the money sent by their families in Sri Lanka. For instance, Sunil, a delivery driver who resigned from his job in early March 2020 intending to return to Sri Lanka, says that he can no longer bear his expenses in Qatar and had to ask his wife in Sri Lanka to pawn her necklace and send him money.

Reverse remittances are not uncommon in the Gulf migration trajectory of Sri Lankan migrants. Migrants who face challenges such as layoffs, non-payment of salaries and various forms of labour exploitations in their host Gulf nations resort to mortgaging or selling their houses, land and other assets to finance their return journey to Sri Lanka. In-depth interviews revealed that such occurrences have become more common with the pandemic as migrants, especially those in low-skill categories, have begun to search for alternatives as they have begun to feel tired waiting for the Sri Lankan government to offer them free or low-cost quarantine options.

In-depth interviews with Sri Lankan migrant workers conducted in September 2020 indicated that compared to semi-skilled workers, skilled and highly-skilled workers who have been laid off or facing delays in payments have been able to weather the storm due to their accumulated savings. For instance, Vimal, the senior manager of the events division of the floriculture company, despite being unable to operate his business for months and not receiving a salary, has been able to bear his living costs in Qatar and the expenses of his family in Sri Lanka from his prior savings. Even though some high-skilled migrants who lost their jobs could not afford to stay in Qatar and look for other job opportunities, they were able to utilise their prior savings to pay for the expensive quarantine process in Sri Lanka and return to Sri Lanka. This spared them from months of the agony of facing financial issues in Qatar. Though they might not be able to find a job immediately upon their return to Sri Lanka, their accumulated savings are likely to help them sustain their lives for a more extended period in Sri Lanka as the cost of living in Sri Lanka tends to be lower than that of Qatar.

However, unlike high-skilled migrants with accumulated savings, semi-skilled migrants, in general, do not have accumulated savings to support them during an extended period of unemployment. Their salaries in the Gulf, in general, are used to purchase consumer durables and to meet everyday household expenses (Athukorala, 1990; Shaw, 2010). For example, for Farooq, the automobile technician who has been without work since April 2020, life in Qatar has been a struggle due to the pandemic. As he had not made a sufficient amount of savings to sustain a long period of joblessness, he was struggling to provide for his family in Sri Lanka, who was depending solely on the remittances he sent.

“Migrating to Qatar allowed me to raise my life from poor category to lower-middle-class category. That’s all I was able to do. I was not able to save. Every month my earnings either matched the spending of my family in Sri Lanka or there was an “overhead”, and I had to borrow. I couldn’t save with that balance sheet. So, now I am faced with great difficulties.”

## A permanent return to Sri Lanka induced by the covid-19 pandemic

As migrant workers to the GCC are guest workers who do not have the option of settling down in their host country, return to their home country is an essential and irrevocable component of the Gulf migration trajectory of Sri Lankan migrants to the region. However, research on highly-skilled migrant workers in Qatar suggests that a significant portion of highly-skilled migrants tend to prolong their stay in the country due to various issues in their home countries as well as the better quality of life in Qatar (Babar et al., 2019; Ekanayake & Amirthalingam, 2020). In-depth interviews conducted for the current research also indicate that migrants of all skill levels, prior to the pandemic, in general, preferred extension of their stay in the country if the opportunity arose. However, both survey results and in-depth interviews suggest that with the spread of the Covid-19 pandemic, a significant proportion of migrant workers across all skill levels are now considering a permanent return.

At the time of the survey in June 2020, nearly 28% of respondents (*N* = 101) were planning to permanently return to Sri Lanka in the coming months, while 51% were yet to make a decision. Only 22% were certain that they would not return to Sri Lanka permanently in the near future. Of the 28 who had made up their minds to return to Sri Lanka, only eight (29%) stated that their return was unrelated to Covid-19. Sixteen (59%) had been planning to return for some time but decided to accelerate their plans due to the pandemic. The other three were returning as they feared that they would be infected with Covid-19 if they continued to stay in Qatar or because they had lost their jobs and were not interested in finding new employment in Qatar.

Even though in June 2020, only 28% of the respondents were planning to return permanently to Sri Lanka, the in-depth interviews conducted in September 2020 indicate that those who are planning a permanent return to Sri Lanka could have considerably increased over the months. While job losses and financial insecurities were the main reason for considering a permanent return to Sri Lanka in the initial months of the pandemic, by September, the main reason for considering a permanent return to Sri Lanka was the trauma of being away from their families in Sri Lanka for an extensive period. Some of these migrant workers who were accelerating their permanent return to Sri Lanka were planning to return to Sri Lanka in a couple of years prior to the pandemic. However, they have decided to accelerate their plans due to the uncertainties caused by the pandemic and the desire to be with their families during these unprecedented and trying times.^6^ This is contrary to the reaction of migrant workers to the GFC, where they continued to reside in their host countries despite reduced employment opportunities and increased financial challenges (Sirkeci et al., 2012).

We found through in-depth interviews that a significant per cent of those considering returning to Sri Lanka permanently due to family separation are high-skilled workers. Unlike low-skilled and unskilled workers who could afford to return to Sri Lanka only once per year or once in two years, even in pre-pandemic times, high-skilled workers who have families in Sri Lanka used to travel between Sri Lanka and Qatar quite frequently prior to the pandemic. However, the length and the cost of the quarantine process and the risks involved with air travel dissuade them from doing so at present. Instead, they consider permanently returning to Sri Lanka to be a better option.

This shows that while, in general, though Sri Lankan migrant workers in Qatar have been able to cope with the financial pressures induced by the pandemic, they struggle with the socio-psychological stresses the pandemic has caused. For those who have not been successful at dealing with either the financial or the psychosocial stresses or have chosen to give up dealing with these stresses, returning to Sri Lanka is an unavoidable path. Such permanent returns, though, would immediately increase the amount of remittances that Sri Lanka receives, could have a negative impact in the long-term.

## Conclusion

This study examined the economic impact of the Covid-19 pandemic on Sri Lankan migrant workers in Qatar. It looked into the type of financial issues migrant workers faced, how the severity of these issues changed over the course of 2020, how these financial issues affected the remittances sent to Sri Lanka and the migrants’ coping mechanisms. A crucial caveat to the findings of this study would be that the sample population does not include any unskilled migrant workers and have very few semi-skilled workers in the survey sample. Therefore, the findings are biased towards the highly skilled and the skilled.

As can be expected, since the onset of the Covid-19 pandemic, a significant portion of Sri Lankan workers in Qatar, akin to other migrant workers across the GCC, has faced numerous financial challenges due to lockdowns and other measures undertaken to contain the spread of coronavirus. Among the various financial issues Sri Lankan migrant workers in Qatar have had to deal with, pay cuts are the most common across all skill levels, with nearly 50% of respondents reporting deductions from their salaries. While reduced pay is a significant financial burden for Sri Lankan migrant workers in Qatar as most of them had migrated to Qatar mainly because of the country’s attractive remuneration packages, in–depth interviews indicate that most Sri Lankan migrant workers consider reduced pay acceptable due to the volatile economic conditions in Qatar posed by the pandemic and because they consider a reasonable pay cut to be far better than being made redundant.

Across the skill levels, there are significant differences in the level and type of financial difficulties faced. As can be expected, the semi-skilled workers have been the most severely affected by the pandemic, with a significant percentage of semi-skilled workers facing difficulties in sending remittances to Sri Lanka, making debt repayments and paying for their food and other basic needs. Compared to highly skilled workers, a higher percentage of skilled and semi-skilled Sri Lankan in Qatar have experienced pay cuts, layoffs, losses in bonus payments and losses in savings due to the pandemic. On the other hand, a higher percentage of highly skilled Sri Lankans in Qatar have experienced losses in gratuity payments and have faced difficulties making rental payments than skilled and semi-skilled workers.

Even though in the early months of the pandemic, the vast majority of Sri Lankan migrants in Qatar expected that their financial position would worsen as the year progresses, with the easing of Covid-19 containment measures in mid-June 2020, the financial situation of some migrants has improved. The most significant improvement is with regard to job security. With a portion of migrants who lost their jobs and those who prefer to be in their home countries during the pandemic leaving Qatar and non-issuance of new work visas due to on-going containment measures, there were more job opportunities available in Qatar by the end of 2020 compared to the early months of the pandemic. Apart from increased job security, for some workers, improved financial stability has been due to the elimination of pay cuts with the easing of lockdowns. It is worth examining whether this improved job security persisted in 2021 with the arrival of new waves of the pandemic.

In-depth interviews revealed that Sri Lankan migrant workers affected by the pandemic use various coping mechanisms to offset or reduce their income losses, such as borrowing from friends in Qatar, moving to accommodations with lower rentals and cutting down food expenses. Some of the coping mechanisms have been extreme, partially due to the severity of the situation that migrant workers anticipated befalling on them as 2020 progressed. It would be interesting to examine whether migrant workers who slashed their expenses due to financial challenges continued to do so or splurged when their financial situation improved with the easing of lockdown restrictions.

In-depth interviews indicated that not all migrant workers have been successful in coping with their financial struggled induced by the pandemic by cutting down expenses. Some of those facing severe financial pressures have resorted to asking their families in Sri Lanka to send them money for the return journey by mortgaging their assets in Sri Lanka. While such reverse remittances are not uncommon among Sri Lankan migrant workers in the GCC, in-depth interviews indicate that the pandemic has increased such occurrences. Further research is required to examine the extent to which the pandemic-led financial challenges led to reverse remittances.

The research findings suggest that migrants who have high levels of accumulated savings have been able to bear the negative impact of the pandemic than those with low levels of or no savings. Semi-skilled migrants who, in general, use their salaries in the Gulf to purchase consumer durables and to meet everyday household expenses in Sri Lanka do not have sufficient accumulated savings to support them during an extended period of unemployment. Therefore, as can be expected, compared to semi-skilled workers, skilled and highly-skilled workers who have been laid off or facing pay cuts and delays in payments have been able to weather the storm due to their accumulated savings.

In-depth interviews suggest that while a significant portion of Sri Lankan migrant workers in Qatar would have preferred to continue to work in Qatar for the foreseeable future prior to the pandemic, with the onset of the pandemic, those willing to continue to stay in Qatar has drastically reduced. For most Sri Lankan migrant workers, while the financial impact of the pandemic was the main reason for considering a permanent return to Sri Lanka in the initial months of the pandemic, by September 2020, the main reason for considering a permanent return to Sri Lanka was the trauma of being away from their families in Sri Lanka for an extensive period.

Therefore, while the easing of Covid-19 containment measures in Qatar has improved the financial stability of most Sri Lankan migrants in Qatar, it might not necessarily transfer into an increase of remittances in the long run as a significant percentage of Sri Lankan migrants are accelerating their plans of leaving Qatar, mostly due to pandemic-related reasons. As a substantial proportion of those contemplating a permanent return to Sri Lanka are highly-skilled migrants who earn higher salaries than other skill categories, the dip in remittances Sri Lanka receives from Qatar could be considerable. This will negatively affect the Sri Lanka economy as a whole as Sri Lanka is a remittance driven economy. However, further research is necessary to determine whether those who were considering returning to Sri Lanka permanently in September 2020 actually returned and whether the pandemic resulted in significant permanent return migration. This is an important distinction that needs to be made as research on prior crises in the GCC (such as the GFC) did not result in significant return migration.

## Data Availability

The dataset used during the current study is available from the corresponding author on reasonable request.

## Footnotes

1 The GCC region includes six nations; Qatar, the United Arab Emirates (UAE), Saudi Arabia, Kuwait, Bahrain and Oman

2 Altruistic remittances refer to transfers made to the country of origin without expectations of anything in return. Remittances arising from self-interest are used by migrants to purchase and maintain assets, expanding social networks that would benefit them upon return or secure an inheritance. Inter-temporal contractual agreements include contractual agreements between the migrants and their families in the home countries that will help one another during difficult periods. (Arun & Ulku, 2011). According to this theory, households in origin countries send one or more family member abroad with the intention of hedging against negative economic situations. However, under these contractual arrangements, migrants remit only if the host country’s macroeconomic volatilities are not related to those of the home country (Emeka Okoro Akpa et al., 2020). Altruistic migrants may increase their transfers to relatives, friends and their communities in their home countries to support them rebuild after a disaster (Bettin & Zazzaro, 2016; Clarke & Wallsten, 2003), while migrants who left their home countries to hedge against these types of adverse conditions remit to maintain smooth consumption and investment in the left behind household (Emeka Okoro Akpa et al., 2020). A self-interested migrant may increase their remittances to their families faced with adverse income shocks if they believe that it would increase their chances of receiving an inheritance (Clarke & Wallsten, 2003).

3 As the Government of Qatar, akin to other GCC governments, do not publish demographic data based on nationality, it is difficult to assess the proportion of Sri Lankans among other nationalities or provide any demographics on Sri Lankan migrants in Qatar. However, estimates by various research groups suggest that Sri Lankans are among the top ten nationality groups in Qatar (Priya Dsouza Communications, 2019).

4 On 5 June 2017, four Arab nations (Saudi Arabia, UAE, Bahrain and Egypt) imposed trade and travel restrictions on Qatari citizens and residents and withdrew their ambassadors from Qatar, leading to a diplomatic crisis in the Gulf region. The blockade was lifted in January 2021, and at the time of writing, the diplomatic ties between Qatar and the blockading countries were slowly progressing toward pre-blockade days.

5 Even though the government of Sri Lanka provided free quarantine facilities in the early months of the pandemic to returning migrants, the capacity of these facilities was far below the required, and the government has been inefficient in arranging alternative options for migrant workers who cannot afford the quarantine fee.

6 The travel restrictions and quarantine rules imposed by both Qatar and Sri Lanka to contain the spread of Covid-19 make it challenging for Sri Lankan migrants to visit their families for short-term visits. In September 2020, Sri Lankan quarantine rules required returning Sri Lankans to quarantine at a designated quarantine facility for a fortnight and then to quarantine at home for another two weeks. In Qatar, returning migrant workers from Sri Lanka were asked to quarantine for two weeks, making the combined quarantine period in Sri Lanka and Qatar six weeks. Most migrant workers cannot afford to take time away from work for a six-week quarantine process.
